# 

**Published:** 1891-03

**Authors:** 


### INDEX.

Abdominal and pelvic surgery, suggestions about, 199.
Abdominal section in a case of cyst of the mesentery, with remarks, 705.
Abdominal sections, thirty-two, unselected; with tables.
Adenoma of pharynx, with cases, 1.
Anæsthetics (Ed.), 561.
Aneurism, circumscribed traumatic, of tibial artery, 9.
Animal heat, sources of, 455.
Animal heat, generation of, 142.
Antipyretics, real and relative value of recent, 129.
Bill, the "doctors" and the Governor's veto (Ed.), 564.
Blenorrhœa, 612.
Book reviews, 51, 190, 320, 382, 448, 499, 509, 575, 576, 640, 704, 767.
Bright's disease (Ed.), 695.
Cancer of the rectum, 274.
Carcinoma lenticulare (selection), 313.
Catarrhal fever, 513.
Clinic reports, 711, 714, 717.
Convulsions, puerperal, when should we interfere in? 207.
Consumption, pulmonary, concentrated food in treatment of, 346.
Correspondence: A card, 687; Letter from Berlin, 546; Letter from J. H. Ridley, M. D., 629; Letter from S. M. Overstreet, M. D., 630; London letter, 359; New York letters, 118, 167, 244, 303, 354, 488, 554, 682, 736, 743.
Croup, how shall we cure? 65.
Cyst, ovarian, an interesting operation for, 223.
Cystotomy, suprapubic, 519.
Drunkenness, is (it) curable? (Ed.) 692
Editorial: 251, 309, 436, 498, 562, 750; Anæsthetics, 561; A text from a sermon, 689; Bright's disease, 695; Doctor's bill and the Governor's veto, 564; Drunkenness, is (it) curable? 691; Georgia Medical Association 123–173; Influenza, the, again, 753; Koch's treatment and other treatments, 309; Koch's treatment, official report of, 174; Legislature *vs.* physician, 366; Logan, Dr. J. P., 368; Medical education, 631; Medical words, 438; Present status of Koch's method, 125; Present status of antiseptic surgery, 126; Quackery, the open door for, 750; Recent graduates, 52; Syphilis, the history of, 635.
Food, concentrated, in the treatment of pulmonary consumption, 346.
Fracture of the cartilaginous portion of the nasal septum, followed by acute traumatic perichondritis, 449.
Gaither, Henry, M. D., In memoriam, 492.
Georgia Medical College agreement, 383.
Gleet and sexual neurasthenia, treatment of, 68.
Gonorrhœa, systemic infection from, synopsis of remarks on, 607.
Gynecology, a plea for conservative minor, 218.
Gynecology, a plea for conservative minor, 257.
Heat, generation of animal, 142.
Hip joint disease, 86 and 142.
Hysteria, report of a strange case of, 392.
Infection, puerperal, 641.
Influenza, the, again (Ed.), 753.
Iritis, syphilitic, 262.
Items, 50, 54, 64, 253, 256, 284, 308, 317, 319, 353, 370, 416, 435, 446, 447, 561, 565, 574, 697.

Kennedy, Dr. L. P. (Ed.), 637.
Koch's lymph, observations on, 193.
Koch, what about Dr., and what about intensely speculative medicine? 135.
Logan, Dr. Jos. P., in memory of, 368.
Malarial hematuria, treatment of, 73.
Medical education, a plea for higher, 14.
Medical education (Ed.), 631.
Medical items, 765.
Medical words (Ed.), 498.
Mercury, 16.
Mercury in the treatment of syphilis, some remarks concerning the use of, 718.
Obstetrics, fifty years' experience in, 333.
Ophthalmia neonatorum, 602.
Ovariotomy during pregnancy, 458.
Perineal section, report of, 717.
Perityphlitis, 214.
Pharynx, adenoma of, with cases, 1.
Pregnancy, ovariotomy during, 458.
Pregnancy, vomiting of, its etiology and treatment, 400.
Puerperal infection, etiology and results, 641.
Quackery, the open door for (Ed.), 750.
Quinine, did (it) kill them? 71.
Selections, 176. Carcinoma lenticulare, 313; The supposed curative effect of operations *per se*, 439, 371, 381, 499–509, 566–573, 637–639, 698, 755–764.
Sinuses, the aseptic closure of long standing, having their origin in tubercular joints, 725.
Skin diseases, notes on the use of sulphur preparations in, 225.
Skin disease, one hundred consecutive cases of, 33.
Skin disease, one hundred consecutive cases of, 103.
Skin disease, one hundred consecutive cases of, 154.
Skin disease, one hundred consecutive cases of, 279.
Skin disease, one hundred consecutive cases of, 349.
Skin disease, one hundred consecutive cases of, 410.
Skin disease, one hundred consecutive cases of, 471.
Skin disease, one hundred consecutive cases of, 515.
Society reports: Alleghany Co. Medical, 285, 420, 527; Atlanta Society of Medicine, 543; Gynecological and obstetrical of Baltimore, 37, 158, 231, 295, 476, 678; Clinical Society of Maryland, 732; Clinical of Louisville, 106; Gynecological of Chicago, 298, 417, 481; Philadelphia Electro-Therapeutic, 47; Southern Surgical and Gynecological Association, 656; Tennessee Medical, 241; Tri-State of Alabama, Georgia and Tennessee, 615.
Sterility in woman, its etiology and treatment, 326.
Stricture of the urethra, organic, the treatment of, by the combined internal and external method, with perineal drainage, 321.
Surgery, abdominal and pelvic, suggestions about, 199.
Syphilis, some remarks concerning the use of mercury in the treatment of, 718.
Syphilitic iritis, 262.
Tetanus, 138.
Tuberculosis, 265.
Tumor, fibroid, two cases of, of uterus, successfully treated by weak currents of galvanism, 468.
Vaginal wall, laceration of the anterior and its repair, 385.
Vomiting of pregnancy, its etiology and treatment, 400.
Yellow fever, 74.

### CONTRIBUTORS TO VOLUME VIII.

Barr, A. D., M. D.......................Calamine, Ark.
Blume, F., M. D.......................Allegheny, Pa.
Bradfield, Jno. R., M. D.......................Alvord, Tex.
Brown, J. Bedford, M. D.......................Alexandria, Va.
Clark, Jno. S., M. D.......................Chicago.
Crichton, L. M., M. D.......................Atlanta.
Fenger, Christian, M. D.......................Chicago.
Fitzhugh, P. H., M. D.......................Atlanta.
Goggans, J. A., M. D.......................Alexander City, Ala.
Howell, J. R. G., M. D.......................Dothan, Ala.
Hutchins, M. B., M. D.......................Atlanta.
Jelks, Jas. T., M. D.......................Hot Springs, Ark.
Johnson, J. C., M. D.......................Atlanta.
Jones, Joseph, M. D.......................New Orleans.
Kime, R. R., M. D.......................Atlanta.
Lamar, Lucius, M. D.......................Atlanta Medical College.
Latimer, J. H., Jr., M. D.......................Atlanta Medical College.
Lockhart, V. O., M. D.......................Homer, Ga.
Lyons, J. A., M. D.......................Chicago.
Mason, B. W., M. D.......................Sheridan, Ark.
Mays, Thos. J., M. D.......................Philadelphia, Pa.
McArthur, L. L., M. D.......................Chicago.
McEvoy, Jos. J , M. D.......................Atlanta.
McKee, E. S., M. D.......................Cincinnati.
McRae, F. W., M. D.......................Atlanta.
Opie, Thos., M. D.......................Baltimore.
Patterson, A. B., M. D.......................Atlanta.
Reeves, Frederick K., M. D.......................Alvord, Tex.
Richardson, E. H., M. D.......................Atlanta.
Rietema, F. A., M. D.......................Rotterdam.
Roy, Chas. D., M. D.......................Atlanta.
Russell, Wm. L., M. D.......................New York.
Small, E. H., M. D.......................Pittsburg, Pa.
Smith, Frank Trester, M. D.......................Chattanooga, Tenn.
Starr, E. F., M. D.......................Nacoochee, Ga.
Stewart, Jno. P., M. D.......................Attalla, Ala.
Stewart, R. W., M. D.......................Pittsburg, Pa.
Szadek, Chas., M. D.......................Kieff, Russia.
Todd, J. S., M. D.......................Atlanta.
Wall, Jno. P., M. D.......................Tampa, Fla.
Wathen, Wm. H., M. D.......................Louisville, Ky.
Watkins, T. J., M. D.......................Chicago.
Westmoreland, W. F., M. D.......................Atlanta.
Williams, Howard, M. D.......................Macon.
Wilson, Augustus, M. D.......................Philadelphia.

